# Public Perceptions of Child Pornography and Child Pornography Consumers

**DOI:** 10.1007/s10508-021-02196-1

**Published:** 2022-01-06

**Authors:** Chad M. S. Steel, Emily Newman, Suzanne O’Rourke, Ethel Quayle

**Affiliations:** 1 Department of Clinical Psychology, University of Edinburgh, Teviot Place, Edinburgh, EH8 9AG UK; 2 Department of Electrical and Computer Engineering, George Mason University, Fairfax, VA USA

**Keywords:** Child pornography, Lay perceptions, Sex offender registration, Stigmatization, Risk assessment

## Abstract

Understanding the public’s perceptions of child pornography helps identify gaps in awareness and knowledge, impacts legislative decision making, quantifies stigmatization, and provides a baseline for identifying differences between lay and offender populations for clinical purposes. This research provides a comprehensive public survey assessing these issues. An Internet-based sample of 524 adults (mean age = 47 years, 51% female) within the USA were asked about their understanding and beliefs related to child pornography and individuals who view child pornography. The questions covered three topic areas—general perceptions of child pornography, endorsement of child pornography beliefs, and opinions related to the legality of various forms of child pornography as well as the decision making related to sentencing and sex offender registration for child pornography consumers. The research found that the public viewed these offenses as more severe than most other crimes and that there was an overestimation by the public of risks related to recidivism and contact offending. Additionally, the research found that there was support for most of the current sentencing guidelines in the USA, including sex offender registration, and that there was limited support for treatment over incarceration.

## Introduction

There has been extensive work done on the public’s perceptions and attitudes related to individuals who have committed sex offenses (Harper et al., 2017), which can influence everything from the acceptance of and access to treatment (Brown, 1999; Wnuk et al., 2006) to public policy related to tracking and registration (Kernsmith et al., 2009; Schiavone & Jeglic, 2009). Instruments such as the Community Attitudes Toward Sex Offenders scale (Church et al., 2008) have evolved and been conceptualized with three factors that have direct relevance to online child pornography consumers, specifically punitiveness, stereotype endorsement, and risk perception (Harper & Hogue, 2015b). This research evaluates the public’s perceptions of child pornography and those who consume it using a similar conceptualization. Punitiveness is evaluated through perceived severity and acceptance of treatment, stereotype endorsement is evaluated by the endorsement of beliefs related to child pornographers, and risk perception is evaluated through estimates of recidivism and contact offending.

The term “child pornography” is used in this research in lieu of the broader term child sexual exploitation material (CSEM) (Frangež et al., 2015). Because child pornography is more familiar to the lay public who were the respondents in this research, and because many of the questions relate to the legal concept which uses that term, this phrasing was used in this paper consistent with the Luxembourg guidelines (Terminology & Semantics Interagency Working Group on Sexual Exploitation of Children, 2016), except where the broader definition is needed to encompass child erotica. In the USA, child pornography is defined by federal law as any visual depiction of a minor (an individual under the age of 18) engaging in sexually explicit conduct (SEXUAL EXPLOITATION AND OTHER ABUSE OF CHILDREN—Definitions, n.d.).

Public perceptions of child pornography have far-reaching consequences, ranging from influences on sentencing guidelines and legislation to contributing to the social stigmatization of child pornography consumers. Despite the far-reaching societal impacts, minimal empirical work has been done to assess the public’s views on child pornography and child pornography consumers. Additionally, there are conflicting social trends that impact those perceptions. General viewing of pornography has become more acceptable and normalized (Diamond, 2009; Dugan, 2018), while views regarding the age of consent show a trend toward increasing rather than lowering the legal consent age (Cawson et al., 2000; Graham, 2018).

The public’s views on child pornography are generally an extension of societal and cultural views on sex offending in general, as well as child sexual abuse, and public policy in this space can be driven by emotion rather than evidence. Child sexual abuse, in particular, may invoke a more visceral emotional response than other sex crimes, with child pornography being an extension of that abuse. In reviewing child pornography laws (primarily in a Canadian and the USA context), Ryder (2003) noted that “Child sexual abuse is a topic that evokes visceral disgust in all reasonable people” (p. 102).

There are several relevant works associated with how the public generally perceives child pornography consumers. Hitikasch et al. (2017), using a German community sample, found that child pornography viewing was rated at the same severity as distribution of child pornography, both of which were viewed as warranting as severe a punishment as sexual assault crimes. Mears et al. (2008) used a telephone poll of adults in the USA that showed the majority of the public support incarceration for child pornography consumers (68%) and that there is significant support for both treatment and the use of sex offender registries for these offenses. Lam et al. (2010) provided lay individuals (Canadian university students) two scenarios to evaluate, varying the age and gender of victims and offenders. They found that lay individuals overestimated the likelihood that a person possessing child pornography was a pedophile, and that they recommended more severe sentencing inversely related to the age of the victim. Additionally, looking at their awareness of the law, 88% of the individuals knew that distribution, and 84% that possession, of child pornography was illegal. Conversely, 45% were unsure that viewing child pornography was illegal and 7% believed it was legal. McCabe (2000) found that most USA citizens polled, 95%, knew that distributing child pornography was illegal, and 92% knew that possessing child pornography was illegal. Conversely, 92% believed that “viewing computer-generated child pornography was okay” (McCabe, 2000, p. 75), and 32% believed that downloading child pornography from a newsgroup was legal. Additional research polling adults in the USA showed support for the illegality of computer-generated child pornography, though at a level that was differentiated from real depictions of children (Kliethermes, 2015), however a survey using a German community sample found that 84% of participants believed that computer-generated sexual imagery of minors should be classified as child pornography (Hitikasch et al., 2017).

Hunn et al. (2020) used vignettes to assess the Australian public’s awareness of legality and their views on victimization. They found high awareness that possession of child pornography is illegal, but limited awareness that possessing virtual child pornography is illegal (possession for virtual child pornography is illegal in both Australia and the USA). In another study evaluating the attitudes of Australian university students, approximately 90% agreed that viewing child pornography had a direct impact on victimization, and 79% agreement that computer-generated child pornography should be illegal (Prichard et al., 2016). Although not evaluating lay individuals, Francis (2015) asked judges and psychologists in the USA their opinions of individuals convicted of child pornography possession, finding that there were strong beliefs in high rates of recidivism and in the ineffectiveness of sex offender registration.

Individuals who possess child pornography are frequently stereotyped as mentally ill or having a “sickness” on the basis of the act itself (US v. Schenberger, 2007; US v. Vanderwerfhorst, 2009). The research, however, provides a picture that is more nuanced. Some studies have found self-reported comorbid psychopathology in the form of personality disorders as high as 40% (Webb et al., 2007), while rates of diagnosed mental illness among child pornography possessors has been shown to be as low as 5% (Wolak et al., 2011). The primary psychopathology associated with individuals who commit child pornography offenses is pedophilia (both as a mental illness and as an undiagnosed exhibition of pedophilic interests), and Seto et al. (2006) found the percentage of individuals who committed child pornography offenses that could be classified as pedophiles to be approximately 60% based on a phallometric response to viewing relevant images.

Recidivism is another area with limited lay understanding that is consistent with distorted risk perceptions (Harper & Hogue, 2015b). Eke et al. (2011) found the 4.1 year recidivism rate for individuals convicted of child pornography offenses committing another child pornography offense to be 6.8%. However, if their only prior conviction was child pornography-related (possession, distribution, or production), the rate dropped to 4.4%. In evaluating their risk assessment tool, CPORT, Seto and Eke (2015) found a similar rate, with individuals who committed only child pornography offenses recidivating at 7% in a 5 year follow-up period. Faust et al. (2015) found an even lower rate of recidivism for individuals who committed only child pornography offenses (1.6%) at an average follow-up time of 4.8 years. Soldino et al. (2020) found a similar 2% rate on a 5 year follow-up with individuals who committed only child pornography offenses. These rates represent re-arrest data and do not include individuals who continued to offend but were not caught again, and thus represent a lower bound approximation.

Another common belief put forward relates to the victimization of those who offend as part of a cycle of abuse. Evidence, however, shows the majority of individuals who committed child pornography offenses had not been sexually abused as a child, with rates ranging from 11.7 (Faust et al., 2015) to 26% (Webb et al., 2007). No comprehensive quantification of the public’s perception of childhood sexual abuse rates among individuals who committed child pornography offenses has been evaluated to-date.

The sexting trend and ubiquity of mobile phones with high quality cameras has changed the dynamics of victimization. Historically, all child pornography represented primary victimization (the sexual abuse or direct exploitation of a child) as well as secondary victimization (the continuance of sexual abuse through repeated distribution and viewing), though self-generated child pornography has altered that pattern (Leary, 2009). Specifically, there may be no primary victimization with self-generated child pornography, excepting cases of coercion or sextortion (Patchin & Hinduja, 2020), and the percentage of self-generated child pornography is growing (Internet Watch Foundation, 2020).

Sentencing and sex offender registration for individuals who commit child pornography offenses is another area of ongoing interest (Christensen & Tsagaris, 2020; Hunn et al., 2018; Proeve & Wolf, 2019), and is influenced by both rehabilitative and punitive attitudes (Harper & Hogue, 2015b). The United States Sentencing Commission provides enhancements that increase the sentences of individuals convicted of child pornography offenses based on viewing habits. These enhancements occur based on the age of the victims portrayed, with an enhancement for possession of images of minors under the age of 12 and a second enhancement if the victim was an infant or toddler. They also occur based on the number of images or videos, and whether or not sadistic/masochistic content is present (United States Sentencing Commission Guidelines, 2018). In addition to traditional sentencing enhancements, many localities require individuals convicted of child pornography offenses to register as sex offenders, despite little evidence of their efficacy and their collateral consequences (Drake & Aos, 2009; Pawson, 2002; Tewksbury, 2005). Public support for these registries remains strong, however, particularly with regard to child sex offenses, indicating a disconnect between perception and efficacy (Kernsmith et al., 2009).

This work empirically measures and evaluates the public’s perceptions of child pornography consumers and child pornography offenses. First, general perceptions of child pornography and child pornography consumers are evaluated. Second, the level of knowledge of the public on various issues surrounding child pornography is assessed against current research. Finally, perceptions associated with the legal implications of child pornography and how child pornography consumers should be evaluated for sentencing purposes is presented. This research represents the most comprehensive study to-date of public perceptions on child pornography and provides results that can be utilized to direct public education on issues related to child pornography to reduce the stigmatization of individuals who commit these offenses and better align public understanding with evidence.

## Method

This work was part of a broader research project looking at the technical behaviors and cognitions of child pornography consumers and consisted of survey questions that were asked of the general public. On the survey, demographic questions and questions related to the respondents’ views and beliefs about child pornography and child pornography consumers were included. The demographic questions were primarily multiple choice and solicited information on the sex, sexual orientation, age, gender, marital status, race, level of education, type of degree, employment status, current occupation, and household income of the participants. The questions related to their beliefs are detailed below.

### Participants

Data were obtained through an anonymous online survey hosted through the University of Edinburgh’s Qualtrics instance. Participants were recruited using the Qualtrics panel service, which provides pre-identified participants from a pool of individuals recruited and compensated by Qualtrics meeting specific criteria outlined by the researcher (*Online Panels: Get Responses for Surveys & Research | Qualtrics*, 2020). Qualtrics panels have been shown to have appropriate representativeness on the dimensions of interest and to be of sufficient quality for research with the appropriate controls in place (e.g., attention and timing checks) (Boas et al., 2020; Miller et al., 2020).

The survey population for this research was English-speaking adults (18 years of age or older) living in the USA. Prior to participation in the survey, panel members were provided with information on how the data collected would be used and both the benefits and risks associated with participation. Participants were required to affirmatively consent prior to starting the survey. Any individuals who chose not to continue with the survey were permitted to withdraw at any point prior to submission, and the results of those individuals were not retained. 624 individuals began the survey, and of those individuals 99 failed to complete the survey and their results were not recorded, resulting in 525 completed surveys.

As part of the survey execution, an initial soft launch with a small number of participants (*n* = 31) was conducted to confirm survey structure and train automated time metrics (as noted below) to address insufficient effort responding (IER) concerns (Kraiger et al., 2019). Additionally, two attention checks were built into the survey, with one as a multiple-choice question and a second as part of a matrix question. Respondents failing the attention checks were automatically discarded by Qualtrics to improve response quality (Owens & Hawkins, 2019). Final completion times in seconds were recorded as part of the soft launch (*M* = 802, SD = 598), and any responses taking less than 203 s (one standard deviation from the mean) were discarded to eliminate individuals answering without taking adequate time to read the questions and responses (*n* = 1). A total of 524 total surveys meeting quality standards were retained for analysis.

The survey participants had a mean age of 47 years and identified their gender as 51% female and 48% male. The participants primarily identified as heterosexual (91%), followed by bisexual (4%) and homosexual (4%). The majority of the participants identified themselves as White or Caucasian (76%), and 15% identified as Black or African American, 8% as Hispanic or Latino, 3% as Asian, 1% as American Indian or Alaskan Native, 1% as Other, and less than 1% as Native Hawaiian or Pacific Islander (participants were permitted to identify in multiple categories). Fifty-two percent of respondents were currently working, and 48% indicated they were in a non-work status, with the majority of those individuals (21%) indicating they were retired. For the highest level of education obtained, 23% completed high school or the equivalent, 24% completed some college but did not receive a degree, 11% received an associate degree, 25% received a bachelor’s degree, and 15% received an advanced degree. Forty-eight percent of participants were married or in a domestic union, 31% were single, 14% were divorced or separated, and 6% were widowed. Participant income ranged from 0 through $15,500,000, with a mean income of $103,091. The principal detailed demographics of the respondents are shown in Appendix Table 2.

### Measures

The questions were broken up into three areas—general perceptions of child pornography and child pornography consumers, endorsement of inaccurate beliefs related to child pornography, and the legality of child pornography and sentencing of child pornography consumers. The overall perception of child pornography possession was measured through a ranking question to allow for comparison to other reference crimes. The questions related to the accuracy of beliefs were enacted using percentage estimates that could be compared to prior research to evaluate their deviation from extant statistics. The questions related to legality were scored using standard 7-point Likert scales to indicate agreement.

#### General Perceptions

To evaluate their overall views on the seriousness of the offense, participants were asked to rank the severity of child pornography possession in relation to other crimes. The reference crimes were taken from the FBI’s Uniform Crime Reporting (UCR) category list, which provides a ranking of crimes by judicial severity (Uniform Crime Reporting Statistics, 2020). The reference crimes, from most severe to least severe, were as follows:

1. Murder and Nonnegligent Manslaughter
2. Rape
3. Robbery
4. Aggravated Assault
5. Burglary (breaking and entering)
6. Larceny-Theft (except auto)
7. Motor Vehicle Theft
8. Arson

The reference crimes, along with child pornography possession, were presented in a randomized fashion to each of the participants, who were asked to rank them in terms of their personal perceptions of severity. The median rankings were then calculated for each of the crime categories.

Data related to the perceived victimization of the minors portrayed in child pornography was ascertained by asking what percentage of those portrayed were willing participants. The participants were additionally asked how difficult it is for individuals who view child pornography to stop, on a 7-point Likert scale ranging from “Extremely Easy” to “Extremely Difficult.”

Two additional questions were asked related to the ease of finding child pornography (to evaluate the public perception of the likelihood of “accidental” viewing) and perceived victimization. In lieu of Likert-type questions where low endorsement was likely, the questions were formulated to capture nuance related to common cognitive distortions (e.g., Virtual is Not Real and Internet is Uncontrollable) (Paquette & Cortoni, 2020; Steel et al., 2020). For the perceived ease of finding child pornography, participants were given four statements related to the likelihood of coming across child pornography and asked which of them they most agreed with:

- Anyone can accidentally come across child pornography while browsing the web.
- Individuals visiting mainstream adult websites may accidentally come across child pornography.
- Individuals visiting less mainstream adult websites may accidentally come across child pornography.
- Only individuals that actively seek out child pornography will find child pornography.

To evaluate the perceived impact of viewing child pornography on child victimization, participants were asked to select which of the following statements they most agreed with:

- Viewing child pornography is directly responsible for creating child victims.
- Viewing child pornography is indirectly responsible for creating child victims.
- Viewing child pornography does not contribute to child victimization (Steel et al., 2020).

#### Endorsement of Child Pornography Beliefs

The participants’ knowledge surrounding the prior sexual victimization of child pornography viewers was evaluated by asking participants to provide a percentage from 0 to 100 (using a slider) of individuals who view child pornography that they believe were sexually abused as children. Similar questions were asked about the percentage of child pornography viewers they believed were pedophiles, what percentage will commit a contact offense against a minor, and what percentage of individuals convicted of viewing child pornography will go on to commit another child pornography-related offense.

#### Legality

In addition to measuring their knowledge, the participants were asked about their views on various aspects of the legality of child pornography, and specific factors related to sentencing within the USA. All items were measured using a 7-point Likert scale, ranging from Strongly Disagree to Strongly Agree. The statements related to the general legality of child pornography and the specific legality of various forms of child pornography were as follows:

- Viewing child pornography is no different than viewing adult pornography.
- Viewing naked pictures of children for artistic (non-sexual) purposes are acceptable.
- Viewing images of naked children where there is no display of the genitals should be illegal.
- Viewing virtual images (lifelike animations and drawings) of children engaged in sexual activity should be illegal.

The additional statements related specifically to sentencing and post-sentencing restrictions were as follows:

- The severity of the acts depicted in child pornography images should be taken into consideration in sentencing decisions.
- Individuals that possess more images and videos should receive longer sentences than individuals with a few images and videos.
- Sentencing of child pornographers should be based on the age of the individuals depicted.
- Individuals who view child pornography should be registered as sex offenders.
- Individuals who view child pornography are mentally ill and should be treated and not put into prison.

### Data Analysis

Exploratory analysis on the results was collected and descriptive statistics presented. Likert scales were displayed using a diverging stacked bar chart, where the vertical line represents the median value (Heiberger et al., 2014). All of the Likert items were scaled between 0 and 6 points, and agreement was considered as any responses of “Somewhat Agree”, “Agree”, or “Strongly Agree.”

To explore demographic associations with the general perceptions of child pornography, independent variables of sex, race, age, income, and education level were examined with the relative severity ranking used as the dependent variable. Sex was analyzed using a Welch’s *t*-test, and race identifications were treated as individual categories with Boolean membership (as individuals could identify with more than one racial category) using individual *t*-tests corrected for multiple comparisons. Age, income, and education level were treated as ranked values and examined using a Spearman correlation. The support for treatment over prison and support for sex offender registration, and the relationships with perceived risk, measured by perceptions of the prevalence of pedophilia, the perceived contact offending rate, and the perceived recidivism rate, were evaluated with Spearman correlations. All the results were collected and analyzed using the R software environment for statistical computing and the RStudio Integrated Development Environment, with a *p* value of 0.01 used for statistical significance tests (where appropriate).

## Results

Overall survey completion dropout rates were low at 16%, likely due to self-selection prior to starting the survey. Additionally, of the individuals who completed the survey, only one did not meet the minimum time requirements for inclusion. This is potentially due to a likely correlation between individuals who did not meet the time requirements and those that failed the attention checks, who were automatically discarded by Qualtrics and not provided to the research team.

### General Perceptions

Child pornography possession was ranked by the general public to be significantly more severe than most other crimes. The median public ranking in perceived severity for child pornography possession was third (after rape and criminal homicide). It was ranked higher than all property crimes and higher than two of the violent crime-against-persons categories, aggravated assault and robbery (Table 1).

**Table 1:** Child pornography possession: Perceived severity rankings

Crime	Median public ranking	FBI ranking
Criminal homicide	2	1
Rape	2	2
Child pornography possession	3	9
Aggravated assault	4	4
Arson	5	8
Robbery	6	3
Burglary (breaking and entering)	6	5
Larceny/Theft (except auto)	8	6
Motor vehicle theft	8	7

Males ranked child pornography possession (*M* = 3.9) as less severe than females (*M* = 3.2), (*t*(500) = − 3.19, *p* = 0.001). Racial group was not significantly correlated with severity, however, those identifying as Hispanic or Latino (*M* = 2.8) ranked possession as more severe (*t*(57) = 2.67, *p* = 0.009) than those who did not (*M* = 3.6). Age and income level were not found to be correlated with severity rating, but a higher degree level was weakly correlated with ranking child pornography possession as less severe (*r*_*s*_ = 0.13, *p* < 0.001).

The majority of respondents believed that, in general, minors were not willing participants (*M* = 23.5, SD = 30.5), with 37% (*n* = 193) believing that minors were never willing participants. The perceived ease of desisting was generally viewed as high, with 67% (*n* = 323) believing that it was at least slightly difficult. Fifty-three percent (*n* = 279) of respondents believed that individuals could come across child pornography without actively seeking it—accidentally in normal web browsing (18%, *n* = 95), when visiting mainstream adult sites (19%, *n* = 99), or when visiting less mainstream adult websites (16%, *n* = 85). Ninety-seven percent (*n* = 510) of respondents believed that viewing child pornography contributed to victimization, either directly (72%, *n* = 375) or indirectly (26%, *n* = 135).

### Endorsement of Child Pornography Beliefs

**Fig. 1:**
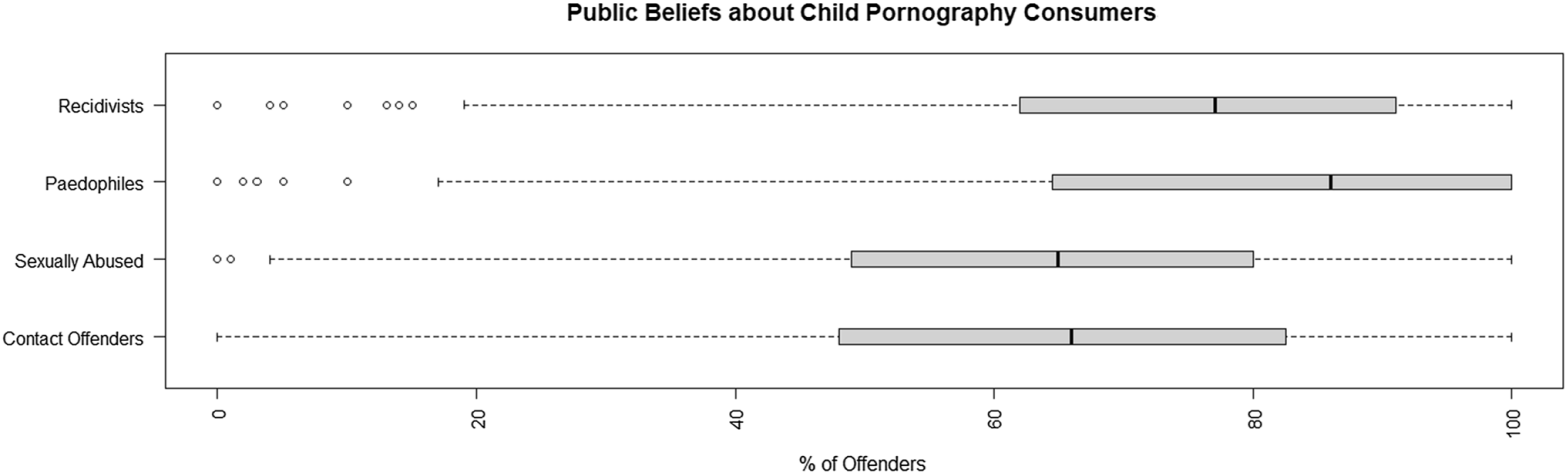
Public perceptions of child pornography consumers

The public perception that individuals who view child pornography were sexually abused themselves was high (*M* = 0.61, SD = 0.24), with a sizable proportion, 13% (*n* = 70), believing that 90% or more of child pornography viewers were abused. Perceptions were similarly high regarding pedophilia, with the sample believing that most child pornography viewers were pedophiles (*M* = 0.79, SD = 0.24), and 42% (*n* = 242) believing that 90% or more were pedophiles. The percentage of individuals who will go on to commit a contact offense was viewed as high (*M* = 0.63, SD = 0.26), with 17% (*n* = 87) believing that 90% or more will commit a contact offense. Recidivism rates were perceived to be high as well (*M* = 0.74, SD = 0.21), with 27% (*n* = 144) believing that 90% or more of individuals convicted of a child pornography offense will commit another child pornography-related offense. A summary of the results is shown in Fig. 1.

#### Legality

**Fig. 2:**
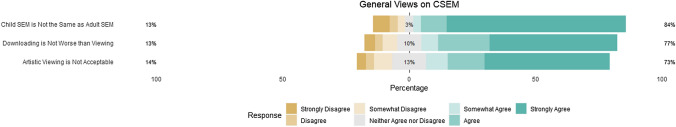
General perceptions of child pornography

**Fig. 3:**
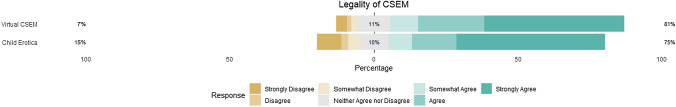
Agreement with child pornography illegality for non-traditional child pornography

In terms of general legality, 84% (*n* = 441) of participants agreed that viewing child pornography was different from viewing adult pornography, 78% (*n* = 406) agreed that downloading was not worse than viewing, and 73% (*n* = 381) agreed that viewing CSEM for “artistic” purposes was not acceptable.^1^ (Fig. 2). Looking at the individual types of CSEM, 81% (*n* = 425) of participants agreed that virtual child pornography (lifelike animations and drawings of children engaged and sexual activity) should be illegal, and 75% (*n* = 393) of individuals agreed that child erotica (images of naked children where there is no display of the genitals) should be illegal (Fig. 3).

**Fig. 4:**
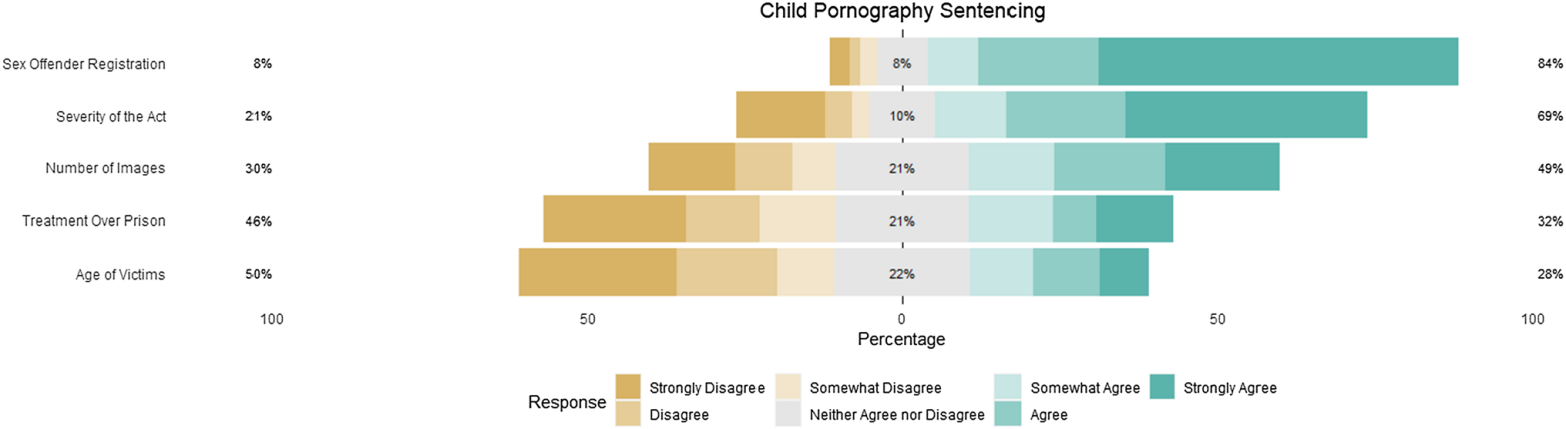
Agreement with child pornography sentencing guidelines and restrictions

For sentencing purposes, there was mixed agreement with the factors that comprise the current sentencing guidelines and restrictions. There was strong agreement for child pornography viewers to be registered as sex offenders, with 84% (*n* = 442) of participants agreeing that sentencing should include registration, and general disagreement for treatment over prison, with only 32% (*n* = 170) supporting treatment. For the specific components that go into sentencing, the overall levels of agreement were mixed, with agreement for the severity of the sexual act being a factor (69%, *n* = 359), but not the number of images (49%, *n* = 257) or the age of the victims (28%, *n* = 148) (Fig. 4).

Support for sex offender registration was moderately correlated with a greater belief that individuals were likely to commit contact offenses (*r*_*s*_ = 0.30, *p* < 0.001), recidivate (*r*_*s*_ = 0.40, *p* < 0.001), and were pedophiles (*r*_*s*_ = 0.41, *p* < 0.001). Support for treatment instead of prison was negatively correlated with a greater belief that individuals were likely to commit contact offenses (*r*_*s*_ = − 0.12, *p* = 0.006), recidivate (*r*_*s*_ = − 0.12, *p* = 0.007), and were pedophiles (*r*_*s*_ = − 0.15, *p* < 0.001).

## Discussion

Members of the public rated child pornography possession as more severe than all property offenses and all but the two most severe offenses against the person, rape and criminal homicide. Male participants ranked child pornography possession as slightly less severe than female participants, which is potentially explained by greater use and acceptance of general pornography by males (e.g., Hald, 2006). The higher severity ranking of child pornography possession associated with identification as Hispanic or Latino is harder to explain, and additional research, potentially incorporating other demographic factors such as religion, is needed to determine the reasons for that difference. There was no perceived difference in severity between viewing and possession, with a very low endorsement of viewing as being a lesser action than downloading and saving child pornography.

There is a current gap in research in evaluating how many minors shown in child pornography were willing participants in the act (separate from the illegal production, distribution, and viewing). Preliminary work indicates it may be a significant minority of images, given the patterns of redistribution of self-generated material (Internet Watch Foundation, 2015; Smith, 2012). Recent data from the Internet Watch Foundation indicated that approximately one third of the reports of web images they had received were self-generated (Internet Watch Foundation, 2020), though what percentage of those individuals were groomed or coerced is unknown and prior research indicates the percentage of self-generated images that involve coercion may be as high as two thirds (Quayle et al., 2018). The public’s perception that the majority of cases involved non-willing participants (which includes minors, where they cannot willingly consent), is reasonable based on historical production mechanisms, the relative recency of the sexting phenomenon, and the amount of coercion present in self-generated images. This is also consistent with the public’s view that child pornography causes direct victimization, which 72% of respondents believed to the most accurate assessment from the choices provided.

More than half of the public sample supported the notion that individuals who view child pornography may come across offending material without actually searching for it, despite a lack of evidence that accidental viewing occurs in practice (Corriveau & Fortin, 2011), showing that this is a common, if inaccurate, belief. The public’s belief in accidental viewing was higher than that in an offender population—Seto et al. found that a large proportion (40% in a police sample and 32% in a clinical sample) of individuals who committed child pornography offenses claimed that they accidentally came across it, though they noted that this explanation was inconsistent with other answers provided by many of those who committed child pornography offenses regarding their motives (Seto et al., 2010). Merdian et al. (2013) identified that approximately 10% of individuals who committed child pornography offenses claimed at least initial accidental access, and Winder et al. (2015) identified similar themes in child pornography consumers’ accounts.

The public’s estimates of the various risk characteristics of child pornography consumers were substantially different than the actual rates, showing high public endorsement of inaccurate child pornography beliefs. For recidivism, the 72% estimate is an order of magnitude higher than the actual recidivism rates, which ranged from 1.6 (Faust et al., 2015) to 2.7% (at 5 years) (Elliott et al., 2019) to 7% (Seto & Eke, 2015). This is consistent with prior research asking university students to predict recidivism, which they predicted to be 69%, and with the public’s view that stopping is difficult for child pornography consumers, which 62% estimated to be difficult (Lam et al., 2010). The perceived risk of contact offending was higher (63%) than the 46% identified in a prior study of university students (Lam et al., 2010), and again an order of magnitude higher than the actual rates, which were measured to be between approximately 3% (Elliott et al., 2019; Faust et al., 2015) and 4% (Seto & Eke, 2005). This is additionally higher than the estimated rate of identified contact offenses at the time of the index offense (12%) as well as the overall self-reported contact offense rate (55%) identified in a previous meta-analysis (Seto et al., 2011), the studies in which primarily looked at individuals who had been arrested for child pornography offenses, representing an upper bound sample. The estimates of the percentage of individuals who commit child pornography offenses that are pedophiles (79%) was also higher than actual estimates of pedophilia of approximately 60% identified by Seto et al. (2006) but consistent with higher estimates from other lay research (Lam et al., 2010). This high estimate additionally shows a potential lack of public understanding of the differences between pedophiles and hebephiles, as well as a general underestimation of other influences that may drive child pornography consumption (e.g., novelty-seeking behavior).

The overestimates of contact offending, recidivism, and the presence of pedophilia were moderately correlated with the support for sex offender registration for child pornography offenses and provide explanatory power for the strong public support for sex offender registries, which was found to be higher (84%) than earlier research (68%) (Mears et al., 2008), despite a lack of evidence of their effectiveness (Bouffard & Askew, 2019). The highest correlation with support for sex offender registration was with perceiving a high prevalence of pedophilia. This is consistent with the general and often vitriolic public dislike of pedophiles—Jahnke et al., for example, found that 28% of an English-speaking sample believed they would be “better-off dead” (Jahnke, et al., 2015a, 2015b). Conversely, support for treatment instead of prison was generally low at 32% and had a weak negative correlation with perceived risk related to contact offending, recidivism, and pedophilia prevalence. Tempering those views, the public estimated that approximately 62% of child pornography consumers were themselves abused as children. This is substantially higher than the actual rates, which were estimated to be approximately 21% (compared to 9% for the reference population) in a meta-analysis (Babchishin et al., 2011).

The results confirmed that there is strong public support for the illegality of virtual or computer-generated child pornography, as well as child erotica, indicating support for revised legislation in this area. As virtual becomes closer to real with improvements in technology, and the understanding that child erotica can be exploited equally by individuals who offend, and as the originalist arguments that the offenses be tied to underlying abuse are less frequent, there is the potential for revisiting the definitions of what constitutes illegal child pornography to include other forms of CSEM. Prior work has shown that individuals did not necessarily tie computer-generated child pornography to direct harm (Hunn et al., 2020), indicating that other factors may be driving the substantial support for illegality, which additional research is needed to elucidate.

For sentencing purposes, there was generally strong support for using the severity of the content in determining sentence length, but mixed support for using the age of victims and the number of images as part of the consideration. This is somewhat consistent with the United States Sentencing Commission guidelines, which allow for a larger enhancement for sadistic and/or masochistic conduct (4 levels) and quantity (up to 5 levels) than age (2 levels) (United States Sentencing Commission Guidelines, 2018)^2^ In particular, the quantity-related enhancement may not be aligned with the viewing of images (as opposed to the possession), which is more difficult to ascertain.

Because most participants are unlikely to personally know someone convicted of possessing child pornography, misperceptions regarding the risk can be, at least in part, attributed to media representation, similar to that for sex offenders as a whole (Harper & Hogue, 2015a). The availability heuristic (Tversky & Kahneman, 1973), provides that the ease with which we recall instances of a rare event can lead to overestimates of the probability that event occurs. The news media have been shown to favor extreme and atypical crimes, especially those involving vulnerable victims, which are recalled when the availability heuristic is engaged. This causes an overestimation of associated risks (O’Connell, 1999). Because child pornography *production* offenses are the most extreme, and because there is a direct victim that can be exemplified, there may be a tendency for individuals to recall these instances more freely and overestimate overall risk (Aust & Zillmann, 1996). Distorted media portrayals may also encourage further victimization. Negative portrayals of mental illness, for example, have been shown to impair help-seeking (Stuart, 2006; Wahl, 1992), and the additional stigma associated with an interest in child pornography may increase offender risk (Seidler, 2010). Additionally, the use of accurate terminology in the media is of high import—the high percentage of the public that perceived individuals who viewed child pornography to be pedophiles indicates a potential conflation of these terms. Encouragingly, prior research has shown that psychoeducation is effective in combating punitive attitudes with other sex offenders and may be of similar benefit in child pornography offenses (Kleban & Jeglic, 2012).

### Limitations

This research was conducted on an English-speaking adult population within the USA, and additional work would be required for generalizability beyond that population. While the quality problems inherent in Internet survey research are well known, the steps taken to validate responses and ensure attention are believed to have minimized these in this research. Additionally, the research was conducted during the 2020 Covid-19 outbreak, which may have influenced unemployment numbers within the demographic data (Coibion et al., 2020).

While attempts were made to use lay terminology, certain clinical terms were included such as pedophilia, whose clinical definition (which requires the attraction to pre-pubescent children) may be more restrictive than the common usage (which may encompass hebephilia). For the ranking criteria, the FBI’s UCR rankings were used as they are already categorized based on severity and used for law enforcement reporting in the USA. Additional research using other child pornography crimes (other than viewing), as well as contact abuse categories, would provide further context on public perceptions of viewing in relation to other crimes against children.

### Conclusions

Providing a comprehensive view of the public’s perceptions on child pornography consumers is critical as an input to both public policy and for clinical purposes. For public policy, this research identified major discrepancies between the perceived risk of child pornography consumers and the actual risk of both recidivism and committing contact offenses. Greater public awareness and targeted education in these areas is strongly needed. These misperceptions can impact legislation related to the illegality of CSEM, sentencing severity and sex offender registration as well as decisions about prosecution instead of treatment, and better-informed decision making is warranted. They can also directly impact the continuing stigmatization of individuals who commit child sex offenses, which is high and provides a barrier to effective treatment and re-integration into society of those individuals (Jahnke et al., 2015a, b; Vitis, 2018).

For treatment purposes, the public’s endorsement of inaccurate beliefs and the explanations they provided for child pornography viewing serve as a baseline for comparison. Additional research looking at how they differ in an offender population can assist in identifying cognitive distortions, which can provide individualized treatment targets. Additionally, the confirmation of the public’s negative views of individuals who view child pornography highlights the need for treatment to include coping strategies for the ongoing stigmatization of these individuals.

## Appendix

See Appendix Table 2.

**Table 2:** Demographics of respondents

Demographic category	Proportion (*n* = 524)
Sexual orientation	
Bisexual	0.04 (*n* = 23)
Heterosexual (straight)	0.91 (*n* = 476)
Homosexual (gay)	0.04 (*n* = 19)
Other	0.01 (*n* = 4)
Prefer not to say	0 (*n* = 2)
Age distribution	
18–24	0.12 (*n* = 65)
25–34	0.18 (*n* = 92)
35–44	0.17 (*n* = 88)
45–54	0.18 (*n* = 93)
55–64	0.16 (*n* = 86)
65 or older	0.19 (*n* = 99)
Gender identity	
Female	0.51 (*n* = 265)
Gender variant/non-conforming	0 (*n* = 1)
Male	0.48 (*n* = 253)
Not listed	0 (*n* = 2)
Prefer not to answer	0 (*n* = 1)
Transgender male	0 (*n* = 2)
Relationship status	
Divorced	0.12 (*n* = 64)
In a domestic partnership or civil union	0.04 (*n* = 21)
Married	0.44 (*n* = 232)
Other	0.01 (*n* = 3)
Separated	0.02 (*n* = 8)
Single, but cohabiting with a significant other	0.05 (*n* = 27)
Single, never married	0.26 (*n* = 137)
Widowed	0.06 (*n* = 32)
Race (Multiple selections permitted)	
American Indian or Alaska Native	0.01 (*n* = 7)
Asian	0.03 (*n* = 18)
Black or African American	0.15 (*n* = 80)
Hispanic or Latino	0.08 (*n* = 43)
Native Hawaiian or Pacific Islander	0 (*n* = 2)
Other	0.01 (*n* = 5)
White or Caucasian	0.76 (*n* = 397)
Employment status	
Not working (disabled)	0.06 (*n* = 33)
Not working (looking for work)	0.08 (*n* = 40)
Not working (other)	0.07 (*n* = 38)
Not working (retired)	0.21 (*n* = 110)
Not working (temporary layoff from a job)	0.05 (*n* = 28)
Working (paid employee)	0.46 (*n* = 240)
Working (self-employed)	0.07 (*n* = 35)
Education level	
Less than high school diploma	0.02 (*n* = 11)
High school graduate (high school diploma or equivalent including GED)	0.23 (*n* = 121)
Some college but no degree	0.24 (*n* = 127)
Associate degree in college (2-year)	0.11 (*n* = 56)
Bachelor’s degree in college (4-year)	0.25 (*n* = 131)
Master’s degree	0.11 (*n* = 60)
Professional degree (JD, MD)	0.02 (*n* = 8)
Doctoral degree	0.02 (*n* = 10)
Income	
$0–9999	0.1 (*n* = 52)
$10,000–20,000	0.11 (*n* = 57)
$20,001–29,999	0.1 (*n* = 52)
$30,000–40,000	0.11 (*n* = 58)
$40,001–50,990	0.12 (*n* = 64)
$50,991–67,000	0.1 (*n* = 54)
$67,001–79,000	0.1 (*n* = 53)
$79,001–100,000	0.1 (*n* = 52)
$100,001–190,000	0.1 (*n* = 52)
Greater than $190,000	0.06 (*n* = 30)

## Footnotes

1 These three scales were reverse coded for consistency of display purposes.

2 Each level indicates a more severe sentence based on tables of offenses.
